# Sphingolipids: drivers of cardiac fibrosis and atrial fibrillation

**DOI:** 10.1007/s00109-023-02391-8

**Published:** 2023-11-28

**Authors:** Junjie Liu, Ximao Liu, Yucheng Luo, Fangze Huang, Yu Xie, Shaoyi Zheng, Bo Jia, Zezhou Xiao

**Affiliations:** 1 Department of Cardiovascular Surgery, Nanfang Hospital, Southern Medical University, Guangzhou, China; 2 Department of Oral and Maxillofacial Surgery, Stomatological Hospital, School of Stomatology, Southern Medical University, Guangzhou, China

**Keywords:** Sphingolipids, Cardiac fibrosis, Atrial fibrosis, Atrial fibrillation, Biosynthesis pathways

## Abstract

Sphingolipids (SLs) are vital constituents of the plasma membrane of animal cells and concurrently regulate numerous cellular processes. An escalating number of research have evinced that SLs assume a crucial part in the progression of tissue fibrosis, a condition for which no efficacious cure exists as of now. Cardiac fibrosis, and in particular, atrial fibrosis, is a key factor in the emergence of atrial fibrillation (AF). AF has become one of the most widespread cardiac arrhythmias globally, with its incidence continuing to mount, thereby propelling it to the status of a major public health concern. This review expounds on the structure and biosynthesis pathways of several pivotal SLs, the pathophysiological mechanisms of AF, and the function of SLs in cardiac fibrosis. Delving into the influence of sphingolipid levels in the alleviation of cardiac fibrosis offers innovative therapeutic strategies to address cardiac fibrosis and AF.

## Introduction

SLs represent a heterogeneous group of lipids that were first discovered in the structural elements of biological membranes and were named after the sphinx due to their perplexing structure [1]. SLs exhibit both hydrophobic and hydrophilic properties and constitute vital constituents of the plasma membrane in virtually all vertebrate cells. In addition, SLs are capable of functioning as signaling molecules that play a role in the regulation of various processes including cell proliferation, apoptosis, adhesion, migration, inflammatory responses, angiogenesis, and intercellular interactions [2–5].

AF is recognized as one of the most prevalent cardiac arrhythmias with an incidence of 1–2% in the general population. According to the 2019 Global Burden Report, AF affects almost 60 million individuals globally and has become a significant public health concern. The prevalence of AF is expected to continue rising globally due to economic growth, an aging population, and the prevalence of risk factors such as diabetes, hypertension, obesity, and alcohol consumption [6, 7].

Although AF is a complex and heterogeneous disease [8], atrial fibrosis is its most prominent feature and is the key factor linking AF-related mechanisms [9, 10]. The main characteristic of atrial fibrosis is abnormal activation, proliferation, and differentiation of fibroblasts, accompanied by excessive synthesis and irregular deposition of extracellular matrix (ECM) proteins [11]. A variety of complex molecular signaling systems are involved in AF, and the development of tissue fibrosis typically occurs in a gradual and progressive manner. Once established, tissue fibrosis is difficult to reverse; therefore, preventing fibrosis by blocking the upstream biological processes that lead to it may be a therapeutic strategy that will benefit patients.

In recent years, the sphingolipid signaling pathway has been recognized as involved in the occurrence and development of fibrosis. Investigating the mechanisms by which sphingolipids participate in cardiac fibrosis can offer new insights for the treatment of cardiac fibrosis and related conditions.

## Structure of sphingolipids

**Fig. 1:**
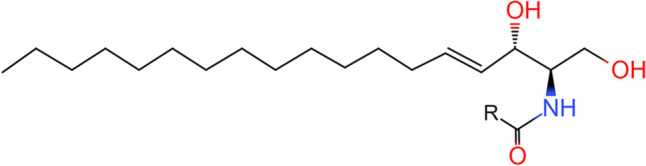
Chemical structure formula of SLs. R: Various fatty acid chains

SLs are a class of amphipathic lipids that consist of a sphingoid base backbone, which is N-acylated with various fatty acid chains and alcohol groups at both ends of the sphingoid base backbone (Fig. 1). This class of lipids includes sphingosine, ceramides, sphingosine-1-phosphate (S1P), ceramide-1-phosphate (C1P) and sphingomyelin (SM). SLs can be divided into three structural types, namely, sphingosine bases and simple derivatives, ceramides and complex SLs, with sphingosine serving as the structural foundation for all sphingolipid derivatives [2].

### Sphingosine and simple derivatives

Sphingoid bases, also known as long-chain bases (LCBs), are non-transient amino alcohol precursors of ceramides and complex SLs. In comparison to complex sphingolipid derivatives, the hydrophilic head group of sphingoid bases consists only of hydroxyl groups. The most common mammalian sphingoid bases include sphingosine ((2 S,3R)-2-amino-4-trans-octadecene-1,3-diol) and dihydrosphingosine ((2R,3 S)-2-aminooctadecane-1,3-diol) which referred to as sphinganine (Table 1). sphingosine is produced via the salvage pathway following ceramide catabolism, whereas sphinganine is synthesized in the de novo biosynthetic pathway. The structural difference between these two sphingoid bases is the presence of a trans double bond at position C4, which is present in sphingosine but absent in sphinganine.

**Table 1:**
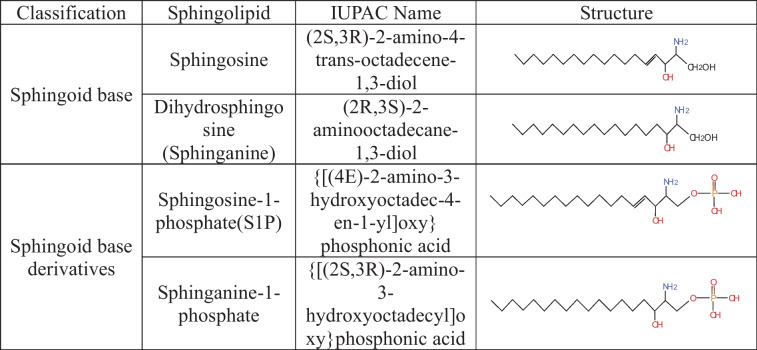
Sphingoid structures of common mammalian

Through minor modifications such as phosphorylation and acetylation, sphingosine can be easily transformed into sphingosine derivatives [12]. Both sphingosine and sphinganine possess terminal hydroxyl groups that can be phosphorylated to form sphingosine-1-phosphate [{[(4E)-2-amino-3-hydroxyoctadec-4-en-1-yl]oxy} phosphonic acid] (S1P) and sphinganine-1-phosphate [{[(2 S,3R)-2-amino-3-hydroxyoctadecyl]oxy}phosphonic acid].

### Ceramides

**Fig. 2:**
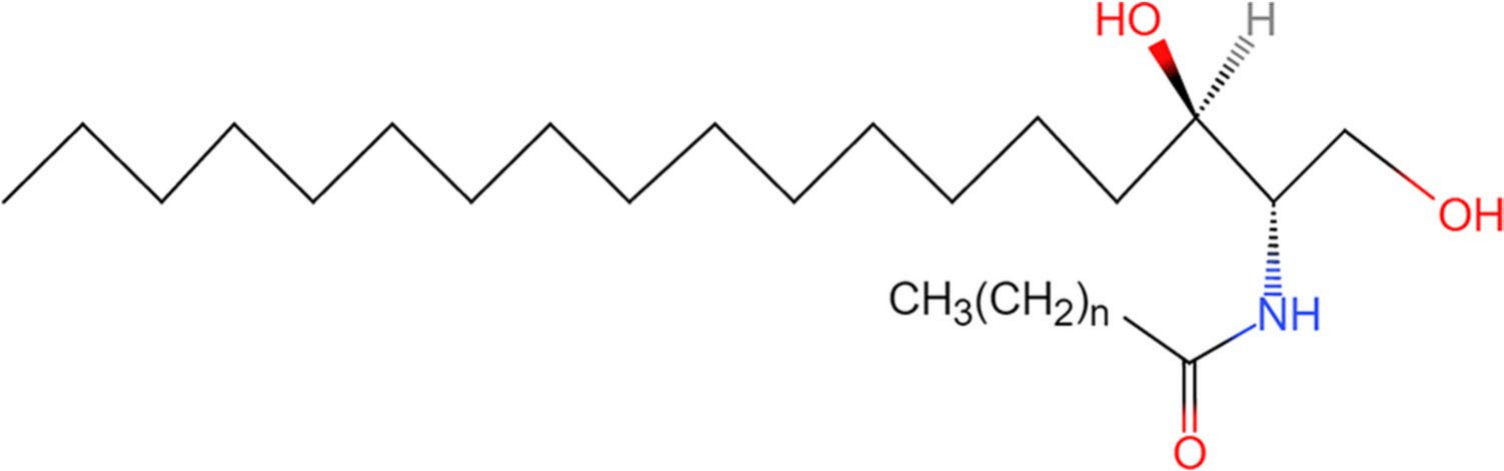
The chemical structure of ceramide

Ceramides are composed of sphingoid bases and variable long-chain fatty acids [13, 14] (Fig. 2). Due to the fact that ceramides possess (1) variable lengths and saturation of the fatty acid chains; (2) the introduction of hydroxyl or double bonds into the sphingoid bases; and (3) the length of the sphingoid base [15], ceramides are not a single substance, but rather a class of structurally similar substances. According to statistics, there may be as many as 360 different ceramide structures [16]. The length, degree of unsaturation, and position of the unsaturated bonds in the fatty acid chains all influence the properties of ceramides. Ceramides are an important constituent of complex SLs, differing from sphingoid bases by the addition of long-chain fatty acids to the amino group. As the backbone of SM, glycosphingolipids (GSLs), and gangliosides, ceramides are essential constituents of the eukaryotic cell membrane. Ceramides also play a critical role as second messengers in cell signaling, and have significant biological functions in cell metabolism, including cell proliferation, apoptosis, and differentiation. This part will be covered in the two chapters of this review on sphingolipid biosynthesis and the association of sphingolipids with cardiac fibrosis and AF.

### Complex sphingolipids

The basic composition of complex SLs comprises a ceramide backbone and a polar head group typically located at the 1-position. Generally, SLs are classified into two major categories based on their head groups: phosphosphingolipids (PSLs) and GSLs; however, these classifications are not mutually exclusive; PSLs can also be considered as acidic GSLs.

#### Phosphosphingolipids

As the name implies, PSLs contain the basic sphingolipid structure, along with one or more phosphate groups. SM is the most common PSL, consisting of a phosphorylcholine and a ceramide, exhibiting a cylindrical structure. SM species constitute the most prevalent SLs in mammalian cells [17] and are a major component of myelin sheaths.

The other PSL is C1P, the main antagonist of ceramide. Despite their similarity, as they differ by only one phosphate group, they perform opposing functions within the cell. Ceramide normally promotes apoptosis, however C1P promotes cell proliferation. This is discussed in more detail in later chapters.

#### Glycosphingolipids

Structurally, GSLs are composed of a ceramide backbone that is covalently linked to at least one carbohydrate moiety. In plants, these carbohydrate moieties are typically simple sugars like glucose, whereas in mammals, they can vary from simple sugars to complex head groups that can be modified by attachment of several carbohydrates or other acidic/neutral molecules. GSLs encompass a vast and varied group of structures that are commonly categorized into neutral and acidic GSLs based on their charge.

## Sphingolipid biosynthesis

**Fig. 3:**
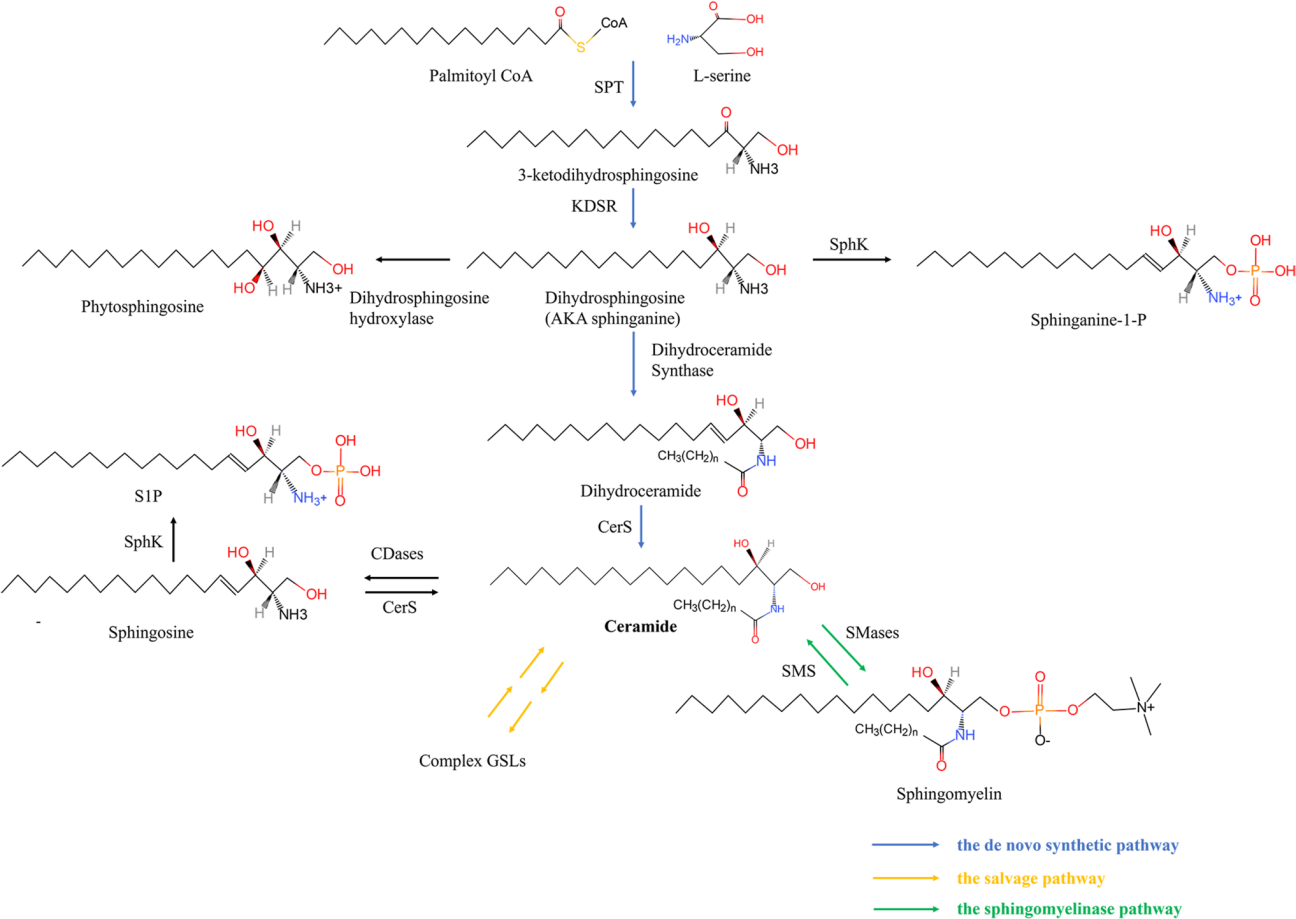
The pathway of ceramide synthesis encompasses the de novo synthetic pathway, the salvage pathway, and the sphingomyelinase pathway. SPT: serine palmitoyltransferase; KDSR: 3-ketodihydrosphingosine reductase; SphK: sphingosine kinases; CerS: ceramide synthase; CDases: ceramidases; SMS: sphingomyelin synthase; SMases: Sphingomyelinases

Ceramide represents a crucial node in the biosynthetic pathway of SLs, and its production is accomplished via three principal pathways: the de novo synthetic pathway, the sphingomyelinase pathway and the salvage pathway [18, 19] (Fig. 3).

### Biosynthesis of sphingoid bases and ceramide via the de novo synthetic pathway

The de novo synthesis pathway initiates within the endoplasmic reticulum (ER) and is accompanied by the decarboxylation and condensation of L-serine and activated fatty acyl coenzyme-A (CoA). Palmitoyl-CoA (C16-CoA) is the most widely employed fatty acyl CoA for sphingolipid production. However, the selection of acyl-CoA substrate is contingent upon the subunit composition of the serine palmitoyltransferase (SPT) enzyme, which catalyzes the condensation reaction. SPT, a pyridoxal 5’ phosphate-dependent enzyme, belongs to the alpha-oxoamine synthase family. This heterodimer consists of two catalytic subunits (SPTLC1, SPTLC2) or a third regulatory subunit (SPTLC3) in place of SPTLC2. Other protein families such as small subunit SPTs (SPTssa and SPTssb) and orosomucoid-like proteins (ORMDLs), perform critical regulatory roles in the SPT complex, resulting in either increased (small subunit SPTs) or decreased (ORMDLs) activity [20, 21]. The selection of specific acyl coenzyme A is determined by the combination of different subunits. The complex SPTLC1/SPTLC2/SPTssa preferentially selects Palmitoyl-coenzyme A (C16-CoA). Moreover, SPTLC1/SPTLC3/SPTssa and SPTLC1/SPTLC2/SPTssb primarily select Myristoyl-CoA (C14-CoA) and Stearoyl-CoA (C18-CoA), respectively [21, 22].

The condensation product of serine and palmitoyl coenzyme A is 3-ketodihydrosphingosine, which is then reduced by NADPH-dependent 3-ketodihydrosphingosine reductase (KDSR). The ketone located at C3 of 3-ketodihydrosphingosine is reduced to an alcohol, resulting in amino alcohol sphinganine (dihydrosphingosine) [23, 24]. sphinganine can be converted into three different derivatives. The first, sphinganine-1-phosphate, is produced by sphingosine kinase via ATP-dependent phosphorylation [25]. The second, dihydrosphingosine, as shown in Fig. 3, can also be converted to phytosphingosine (4-hydroxysphinganine) by adding a hydroxyl group to C4.

The third biological derivative, dihydroceramide, as shown in Fig. 3, arises from the continuation of the de novo synthetic pathway. Dihydroceramide synthase, often referred to as ceramide synthase (CerS), catalyzes the attachment of the acyl group of fatty acyl-CoA to the free amino group of sphinganine via an amide bond, producing dihydroceramide [26]. There are six enzymes in the CerS family that have been identified, each with a specific preference for the length of acyl-CoA chain used for N-acylation of the sphingoid LCB (Table 2). CerS1 was the first CerS to be discovered due to its homology to Lag1 in yeast and demonstrates a preference for C18-CoA. CerS2 prefers C22-C24-CoA, CerS3 utilizes C26-CoA and higher CoA, CerS4 utilizes C18-C20-CoA, and CerS5 and CerS6 utilize mainly C14-16-CoA [22, 27–30]. The dihydroceramide produced by these sphingosine N-acyltransferases is then dehydrated by dihydroceramide desaturase with the addition of a 4,5-trans double bond, resulting in ceramide [31].

**Table 2:** Specificity of different CerS to acyl-CoA

Ceramide synthase	Tissue distribution	Acyl-CoA specificity	Pathologies associated with increased ceramide synthase activity
CerS1	Brain, skeletal muscle	C18:0	Skeletal muscle insulin resistance
CerS2	ubiquitous	C22:0	Cardiac mitochondrial dysfunction
		C24:0	Neutral/benign
		C24:1	Cardiac mitochondrial dysfunction
CerS3	Skin, testes	≥C26:0	Farber disease biomarker
CerS4	Heart, lungs	C18:0	Unknown
		C20:0	Lung cancer, heart failure
CerS5	ubiquitous	C14:0	Unknown
		C16:0	Heart failure, apoptosis
CerS6	ubiquitous	C14:0	Unknown
		C16:0	Heart failure, apoptosis, adipose tissue dysfunction, liver insulin resistance and liver fibrosis

### The salvage pathway and the sphingomyelinase pathway

As illustrated in Fig. 3, complex SLs such as GSLs, are partially degraded and their respective components are recirculated to form ceramides, which is known as the salvage pathway. Sphingomyelinases (SMases) catalyze the hydrolysis of SMs to generate ceramide and phosphorylcholine, known as the sphingomyelinase pathway [17]. GSLs are transported from the plasma membrane to lysosomes by cytocytosis and degraded by specific enzymes with the assistance of accessory proteins [32, 33]. The glycan fraction of GSLs is removed, leading to the formation of ceramides, which are subsequently deacylated by ceramidases (CDases) to produce sphingosine and free fatty acids. As mentioned previously, sphingosine is only produced in the salvage pathway through the complex hydrolysis of SLs and ceramides. Sphingosine can be phosphorylated by sphingosine kinases (SphK1 & SphK2) to produce S1P. Alternatively, sphingosine can be translocated to the ER, where it is reused for ceramide formation via CerS, subsequently producing complex SLs [34].

### Formation of complex sphingolipids

Complex SLs are synthesized by attaching hydrophilic head groups to the hydroxyl groups located at C1 of a hydrophobic ceramide. As previously mentioned, they can be categorized into two categories: PSLs and GSLs. Ceramides produced by the de novo synthetic pathway or the salvage pathway are the basis for all complex SLs.

#### Phosphosphingolipids

PSLs are formed by attaching a phosphate-containing polar head group to the ceramide parent compound. In the case of SM, the ceramide parent compound is phosphocholine [35]. After completion of synthesis in the ER, ceramide is transported to the Golgi inner leaflet via ceramide transporter protein (CERT) [32, 36, 37]. The head group of a phosphorylcholine is transferred from the phosphatidylcholine to the ceramide via sphingomyelin synthase (SMS), producing diacylglycerol (DAG) and SM (ceramide phosphocholine) [17]. There are three SMSs (SMS1, SMS2 and SMSr), which are encoded by the genes SGMS1, SGMS2 and SAMD8. SMS1 and SMS2 each have six transmembrane structural domains and perform the same catalytic function but are located at different sites; SMS1 is found in the trans-Golgi apparatus, whereas SMS2 is in the plasma membrane [17, 38].

As a substitute for the choline used to form SM, ethanolamine can be used as the phospho-alcohol fraction, supplied by a phosphatidylethanolamine to produce ceramide phosphoethanolamine (CPE). A less active homologue of SMS, called sphingomyelin synthase-related protein (SMSr), preferentially utilizes phosphatidylethanolamine as a donor, resulting in the production of CPE [39]. SMSr is a six-transmembrane protein similar to its SMS counterpart, although it is located within the lumen of the ER [40].

Sphingosine produced in the salvage pathway is phosphorylated by sphingosine kinase at the C1 hydroxyl group to form S1P. This process takes place in various cellular compartments including the plasma membrane, mitochondria, nucleus, and lysosomes. There are two isoforms of sphingosine kinase, SphK1 and SphK2. SphK1 is predominantly located in the cytoplasmic lysis and phosphorylates sphingosine from the lysosomal cytosol to form S1P. However, SphK1 can move to the plasma membrane to be phosphorylated by extracellular signal-regulated kinase 1/2 (ERK1/2), where it also forms S1P with sphingosine in the membrane [40]. On the other hand, SphK2 predominantly localizes in the nucleus and mitochondria [36, 41, 42].

C1P is produced through direct phosphorylation of ceramide by ceramide kinase (CERK), which is predominantly generated in the trans-Golgi network but is also detected in the nucleus and plasma membrane. CERK contains an N-terminal myristoylation site and pleckstrin homology domain that It is used for cell membrane binding [36, 43]. Moreover, CERK belongs to the DAG kinase family. CERK selectively recognizes ceramides containing sphingosine and exhibits greater affinity towards those with acyl chains greater than 12 carbons [17]. Once synthesized, C1P is transported to the plasma membrane via the ceramide phosphate transfer protein [36].

#### Glycosphingolipids

GSLs are formed through the combination of hydrophobic ceramide groups and hydrophilic carbohydrate head groups and can be broadly classified as neutral or acidic GSLs based on their carbohydrate composition. neutral GSLs are also commonly referred to as cerebrosides.

**Fig. 4:**
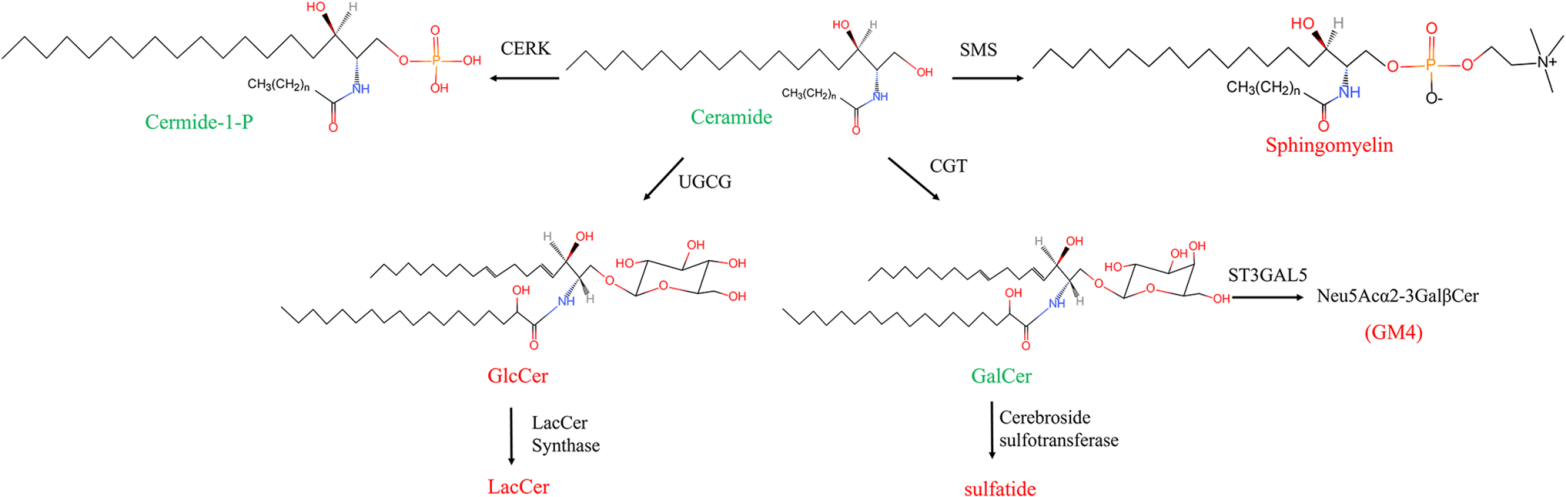
Biosynthesis of complex SLs. SLs synthesized in the endoplasmic reticulum are in green and those synthesized in the Golgi apparatus are in red. Ceramides are the main branching point in the biosynthetic pathway of various SLs, including SM, C1P and simple GSLs such as GlcCer and GalCer. UGCG is responsible for the addition of glucose molecules to ceramides, while CGT adds galactose molecules to ceramides. GalCer can be sialylated by the sialyltransferase ST3Gal V to produce GM4. In addition, GalCer may be sulfated by cerebroside sulfotransferase to form sulfatide. GlcCer is converted to LacCer by the addition of Gal onto the Glc headgroup. CERK: ceramide kinase; SMS: sphingomyelin synthase; UGCG: UDP glucose ceramide glucosyltransferase; CGT: ceramide galactosyltransferase

As shown in Fig. 4, once formed in the ER, ceramides can be galactosylated by ceramide galactosyltransferase (CGT), a type I transmembrane protein, using uridine diphosphate galactose (UDP-Gal) to form Galactosylceramide (GalCer) on the luminal surface of the ER [32, 44]. GalCer can then be transported to the Golgi apparatus for further modification, for example by the addition of sulphate to the C3 hydroxyl group, converting it to sulfatide, or it can be sialylated by the sialyltransferase ST3GalV to form Neu5Acα2-3GalβCer (GM4) [17]. Additionally, ceramide can reach the Golgi complex via one of two transport pathways: (1) transport via CERT, which transports ceramide to the trans-Golgi network to form SM; or (2) vesicular transport to the cis-Golgi network, which is used to produce glucosylceramide (GlcCer) via glycosylation [32, 37]. The formation of GlcCer is mediated by enzyme UDP-glucose ceramide glucosyltransferase (UGCG), which transfers a glucose moiety taken from activated UDP-glucose to the hydroxyl group at C1 of the ceramide in β-linkage (O-linked glycosylation) [45, 46].

GlcCer is transported via the Golgi apparatus and can be galactosylated by β4 galactosyltransferases V and VI to form LacCer, which becomes a branching point for the addition of more monosaccharides to form globular glycosides (neutral GSL) or the addition of one or more acids and subsequent formation of acidic GSL [46].

## The pathophysiology of AF

**Fig. 5:**
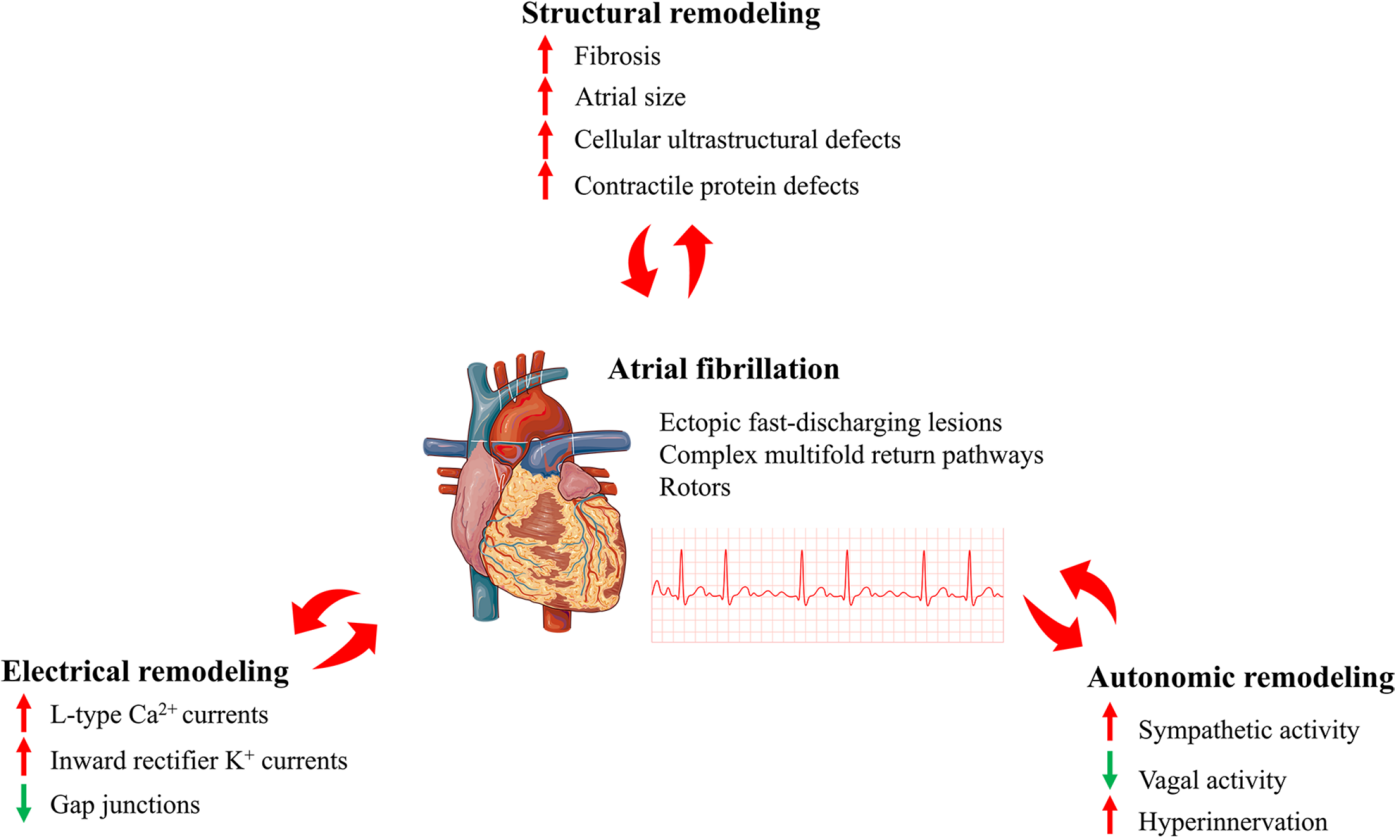
Main types of remodeling leading to atrial fibrillation [9]

The main ECG manifestations of AF are the absence of P waves and the presence of irregular ventricular rhythms without repetitive patterns. Clinically, AF can be defined as paroxysmal (converted to normal sinus rhythm within 7 days), persistent (converted to normal sinus rhythm after 7 days), long-standing persistent (lasting for more than 12 months), or permanent (unable to be terminated and converted to normal sinus rhythm) [47]. Current research and exploration of AF support the hypothesis that AF is produced by the interaction between a ‘trigger’ (initiating electrical stimulation) and a ‘substrate’ (vulnerable tissue causing AF to be induced and sustained in certain cases) [48]. The development and persistence of AF requires pathophysiological remodeling of the atria. Regardless of whether it is a simple AF or a secondary effect of other cardiac diseases, changes associated with AF remodeling can be classified into three categories: (i) electrical remodeling, involving modulation of L-type Ca^2+^ currents, various K^+^ currents, and gap junctional function; (ii) structural remodeling, entailing alterations in tissue properties, size and ultrastructure; and (iii) autonomic remodeling, including altered sympathetic vagal activity and hyperinnervation [49]. As the result of pathophysiological remodeling of the atria, complex electrical defects are created in the atria, including foci of ectopic rapid discharge, complex multifold return pathways or rotors [50]. This will therefore increase susceptibility to AF, leading to its induction and perpetuation (Fig. 5) [51].

Atrial fibrosis is the most prominent feature of atrial remodeling in AF [52]. Although there is controversy about whether atrial fibrosis is the cause or just a consequence of AF, numerous studies have suggested that fibrosis is the cause of AF and that AF further exacerbates fibrosis. First, many animal models of atrial fibrosis have shown that atrial fibrosis increases susceptibility to AF [51, 53, 54]. Several specific pro-fibrotic signaling molecules including angiotensin II (Ang II), aldosterone and transforming growth factor-β1 (TGF-β1) are associated with atrial fibrosis and AF [55–57]. In animal models, AF can be prevented by preventing fibrosis [58, 59]; secondly, in the absence of any abnormalities in cell electrophysiology, atria fibrosis was observed in a transgenic mouse model of isolated atrial fibrosis and can induce AF [60, 61]; finally, studies have also demonstrated that fibroblast function can be activated during rapid atrial pacing [62].

Atrial fibrosis is characterized primarily by abnormal activation, proliferation and differentiation of fibroblasts, as well as excessive synthesis and irregular deposition of ECM proteins, the essence of which is an imbalance between collagen synthesis and its catabolism [11]. The ECM of the heart consists mainly of fibrillar type I collagen (accounting for approximately 85% of total myocardial collagen) and type III collagen (accounting for around 11% of total myocardial collagen). Type I collagen is mainly associated with thick fibers that have tensile strength. In contrast, type III collagen usually forms thin fibers that maintain the elasticity of the matrix network [63, 64]. In addition to collagen, the ECM of the heart contains glycosaminoglycans (such as hyaluronic acid), glycoproteins and proteoglycans as well as a large number of potential growth factors and proteases, whose activation after cardiac injury may trigger a fibrotic response. Fibrosis will maintain the integrity of the heart; however, scar proliferation formed by collagen following fibrosis interferes with electrical signaling [65].

**Fig. 6:**
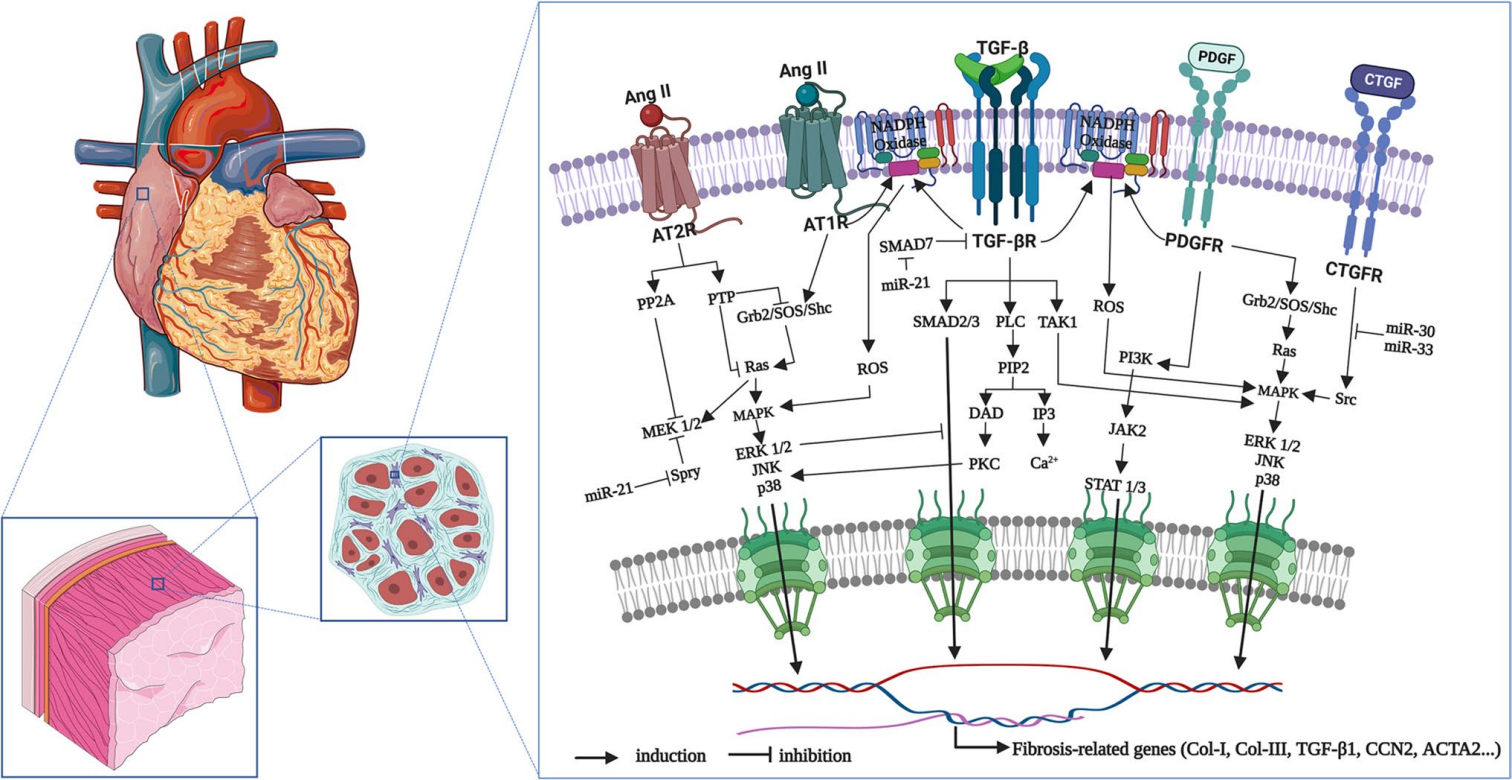
Signaling pathways associated with atrial fibrosis

Atrial fibrosis is a complex process that involves various intricate molecular signaling systems (Fig. 6). The major pro-fibrotic cell membrane receptor factors identified to date include connective tissue growth factor (CTGF), Ang II, platelet-derived growth factor (PDGF), and transforming growth factor-β (TGF-β) [66]. The downstream signaling pathways involves multiple common intermediates that increase the production of ECM proteins through a range of transcription factors as well as signaling molecules that exert positive feedback on the fibrotic process. Meanwhile, inflammation and many inflammation-related cytokines and cellular mediators have also been reported to release and activate pro-fibrotic molecules to cause fibrosis [67].

## Association of sphingolipids with cardiac fibrosis and AF

SLs, heretofore, have been ascertained to participate in the fibrotic processes of various organs, including the liver, lungs, kidneys, and ocular structures [68–70]. Notably, SLs serve as discernible markers for hepatic fibrosis and inflammation. A study has proffered evidence indicating a noteworthy correlation between plasma S1P levels and the mortality rate of the Model for End-Stage Liver Disease [71]. Activated hepatic stellate cells, reliant upon the enzymatic action of SphK, instigate hepatic fibrogenesis. The TGF-β signaling pathway, when activated, orchestrates an upregulation of SphK1 expression, culminating in an elevation of S1P levels and a concurrent reduction in ceramides. This cascade of events precipitates the differentiation of hepatic stellate cells into myofibroblasts [72]. Hao et al. have found the inhibition of S1P-S1PR_1_ signaling to provoke an exacerbation in pulmonary fibrosis [73]. In the realm of diverse chronic renal pathologies, SLs tend to accumulate within the renal milieu, thereby precipitating renal fibrosis and, ultimately, renal insufficiency [69]. Furthermore, S1P exerts its influence on processes involving the proliferation of retinal pigment epithelial cells, differentiation of myofibroblasts, and the synthesis of collagen, thereby catalyzing ocular fibrogenesis. Notably, these processes can be ameliorated through the application of anti-S1P antibodies [74, 75].

Gonzalez-Cordero et al. analyzed the genes from patients with AF and found a number of single nucleotide polymorphisms significantly associated with AF, some of which encode proteins involved in lysosomal activity that break down ceramide into sphingosine and lead to collagen deposition around atrial cardiomyocytes [76].

Biologically active SLs are now considered to possess a diverse range of functions that involve almost all major aspects of cellular biology, including cell proliferation, cell regulation, cell adhesion, cell migration, inflammatory responses, angiogenesis and intercellular communication [2–5].

### The relationship between ceramide and cardiac fibrosis

Ji et al. discovered that reducing cardiac and plasma ceramide levels decreased ventricular remodeling and fibrosis in a mouse model of heart failure (HF) induced by ischemia. The researchers induced myocardial infarction in mice by ligating the left anterior descending branch of the coronary artery, producing left ventricular dysfunction and progressive cardiac remodeling and dilatation. In the HF with reduced ejection fraction (HFrEF) model, application of Myriocin (an inhibitor of SPT) reduced cardiac ceramide levels, as well as ventricular remodeling and fibrosis in the HFrEF mouse model. Similar results were also observed in Sptlc2^+/−^ mice (lacking the Sptlc2 subunit) [77]. Reducing cardiac and plasma ceramide levels by promoting ceramide degradation through CDases can similarly reduce cardiac remodeling and fibrosis and thus improve cardiac function. Adiponectin is an adipose-derived hormone that promotes weight loss, increases insulin sensitivity, reduces inflammation and inhibits apoptosis and has anti-diabetic and cardioprotective effects [78]. Adiponectin was found to increase the intrinsic CDases activity of its two receptors, AdipoR1 and AdipoR2 [79, 80]. Inhibition of acidic CDases activity in mice after myocardial infarction was found to exacerbate the impairment of cardiac function, while an increase in acidic CDases activity improved cardiac function [81], suggesting an important role for the CDases response in HFrEF. These studies provide evidence that ceramide acts as a cardiotoxin that impairs cardiac function and suggest that the application of interventions to reduce ceramide levels may have a protective effect on the heart.

Ceramide has a role in regulating cellular apoptosis and senescence [82], while ceramide can also lead to neuroinflammation via apoptosis. De Wit et al. found that elevated levels of ceramide in reactive astrocytes promote neuroinflammation [83]. The accumulation of ceramide can induce inflammatory oxidative stress, leading to cystic fibrosis and emphysema [84]. Studies conducted on animals have shown that apoptosis in the context of fibrosis may contribute to the pathophysiological profile of AF [85–89], and ceramide may also play a role in atrial fibrosis and remodeling through oxidative stress and inflammatory responses [36, 62, 90–92].

### The relationship between C1P and cardiac fibrosis

In addition to regulating the growth of primary photoreceptor progenitors, primary bone marrow-derived macrophages (BMDM), C2C12 macrophages and various cancer cell types, C1P also stimulates DNA synthesis and cell proliferation in fibroblasts.

The stimulation of cell proliferation by C1P involves multiple signaling pathways. For example, in BMDM, C1P activates the ERK, c-Jun N-terminal kinases (JNK) and PI3-K/PKB pathways, leading to the phosphorylation of NF-κB and GSK-3β. This culminates in upregulation of cyclin D1 and c-Myc, thereby stimulating macrophage proliferation [93, 94]. Furthermore, the phosphorylation of the mammalian target of rapamycin (mTOR), specifically the kinase complex (mTORC1), has been found to be a key step in the mechanism of C1P-stimulated macrophage proliferation [95]. Fibroblasts can change from a quiescent to a proliferative, migratory, secretory state in the context of myocardial infarction, altering their phenotype to become myofibroblasts, which then express contractile proteins, including α-SMA, ultimately leading to myocardial fibrosis [96].

Moreover, C1P is involved in the inflammatory response and can enhance phospholipase A2 (PLA2) activity, thereby promoting the release of arachidonic acid (AA) from membrane phospholipids [97]. Pettuss et al. found that C1P regulates the inflammatory response by activating the synthesis and release of AA and prostaglandins, and they also discovered that CERK, which produces C1P, is an upstream regulator of PLA2 activation [98]. Inflammation plays an important role in fibrosis and remodelling following cardiac injury [36, 62, 90–92], implying that C1P may regulate the inflammatory response and, consequently, participate in cardiac fibrosis.

### The relationship between S1P and cardiac fibrosis

S1P plays a crucial role in tissue fibrosis [99, 100]. Its concentrations in tissues and/or plasma correlate with a number of fibrotic factors, including TGF-β, PDGF and CTGF [101]. Increased concentrations of S1P in peripheral blood are also observed in a variety of fibrosis-related diseases, whereas blocking of S1P with antibodies against S1P is effective to reduce TGF-β-mediated collagen production [102]. Relaxin (RLX), a peptide hormone that causes physiological cardiac effects, is a key regulator of ECM remodeling in many tissues [103]. A research using immature primary cardiomyocytes isolated from neonatal mice and mouse cardiac fibroblasts H9C2 has suggested that RLX mediates SM metabolism, SphK1 activation, and S1P production in cardiomyocytes. Moreover, RLX-mediated S1P production is critical for ECM remodeling in cardiomyocytes [104].

Both intracellular and extracellular S1P can impact cell growth and survival through multiple pathways involved in fibrotic activity [105]. S1P possesses pro-proliferative and anti-apoptotic properties and can act as an antagonist of ceramide mediated apoptosis by activating ERK and inhibiting ceramide-induced activation of JNK [106, 107].

S1P can be involved in inflammatory responses. Yogi et al. found that S1P promotes activation of p38MAPK and JNK/SAPK and induces inflammatory mediator production, in addition to stimulating inflammatory pathways through S1P1 receptor-mediated tyrosine kinase phosphorylation. This response was amplified in spontaneously hypertensive stroke-prone rats, possibly due to increased phosphorylation of PDGF and epidermal growth factor receptors. This research indicates that S1P may induce pro-inflammatory signaling pathways that could affect hypertensive vascular inflammation [108]. Furthermore, elevated pro-inflammatory responses, including elevated pro-inflammatory cytokines (IL-23/IL-17/G-CSF cytokine axis), increased expression of inflammatory product genes, and higher levels of blood neutrophils and monocytes were found in S1P lyase knockout mouse models due to S1P accumulation [109]. Additionally, during myocardial hypoxia, S1P plays an important role in the pro-inflammatory response and migration of cardiac fibroblasts [110]. S1P can also enhance the expression of cyclooxygenase 2, promoting an increase in PGE2 [97]. In contrast, Fettel et al. demonstrated that S1P inhibited leukotriene biosynthesis in neutrophils by inducing S1PR_4_-mediated Ca^2+^ mobilization, suggesting that S1P has anti-inflammatory effects [111].

It has been demonstrated that S1P plays a role in tissue fibrosis possibly through the regulation of autophagy [112].

S1P signaling can regulate fibroblast migration and myofibroblast differentiation, vascular permeability and TGF-β signaling through its receptors and is associated with fibrotic responses to tissue injury [113–118]. S1P receptors (S1PRs) are crucial components in various life processes. S1PR_1_ has been found to play a crucial role in embryonic angiogenesis [119] and is also known to regulate blood pressure in adult individuals [120]. S1PR_2_ has been shown to be involved in the morphogenesis of the zebrafish heart [121]. In vivo, the expression of S1PRs is regulated by a variety of factors and cytokines. TGF-β1 has been shown to be a potent regulator of S1PRs expression [122]. Additionally, the stimulation of human macrophages using supernatants from cultured apoptotic cells can increase the expression of S1PR_1_ in macrophages [123]. Increasing evidence supports an important role for S1PRs in the fibrosis process in various cells [100]. A study using S1PR_2_-deficient mice or S1PR_3_ and S1PR_2_ double-deficient mouse models found that the activity of Rho, which is closely associated with fibrosis, was significantly reduced in these mouse embryonic fibroblasts [124]. Furthermore, inhibition of S1PR_2_ and S1PR_3_ gene expression in vitro led to the inhibition of myofibroblast differentiation, and this study also indicated that S1PR_2_ and S1PR_3_ are localized to the cell membrane [125].

S1P-S1PR_3_ signaling has been found to promote cardiac fibrosis in addition to liver fibrosis. Mice overexpressing sphingosine kinase 1 (SphK1), the enzyme responsible for producing S1P, were observed to develop spontaneous cardiac fibrosis. When these mice were crossed with S1PR_3_-deficient mice, the ability of SphK1 overexpression to produce cardiac fibrosis was diminished, suggesting that the pro-fibrotic effects of S1P in the heart are at least partially mediated by S1PR_3_ [126]. Furthermore, S1P-S1PR_2_ signaling has been demonstrated to induce increased differentiation of cardiac fibroblasts and collagen production in rats [102].

S1PR_1_, S1PR_2_ and S1PR_3_ are expressed in the heart. The distribution of S1PR isoforms varies depending on the type of cardiomyocyte: S1PR_1_ is mainly expressed in cardiomyocytes with lower levels of S1PR_2_ and S1PR_3_ [127, 128], whereas S1PR_3_ is mainly distributed in fibroblasts [129]. A study showed that S1PRs are critical in regulating myocardial function as they control ion channels and mediate myocardial self-protection during ischemic preconditioning [130]. In mouse experiments, S1PR_1_ expression is upregulated during myocardial hypertrophy and can lead to myocardial hypertrophy and fibrosis by inducing interleukin (IL)-6 secretion in a manner dependent on Ang II-AT1, though this occurs only in proliferating fibroblasts and not in cardiac myocytes [131]. Conversely, activation of S1PR_2_ and S1PR_3_ in vivo attenuates myocardial ischemia-reperfusion injury, potentially via S1P-mediated ventricular fibrosis affecting fibroblast differentiation into myofibroblasts [132, 133]. Furthermore, knockdown of S1PR_3_ in cardiac cells can inhibit fibrosis in SphK1-high expressing mice through Rho- and Smad3-dependent signaling pathways [126]. FTY720, an S1PRs agonist, can produce potent anti-inflammatory and antioxidant effects by inhibiting oxygen free radicals, thereby reducing cardiomyocyte death and formation of myocardial fibrosis [134]. FTY720 is also an effective ischemic preconditioner, as demonstrated by a study using a mouse model of heart transplantation, where it reduced apoptosis, inflammation, and oxidative stress, thereby ameliorating myocardial fibrosis [135].

Expression of SphK1 in cardiac fibroblasts can affect cardiomyocyte degeneration and fibrosis. In vivo studies have shown that SphK1 transgenic mice develop myocardial degeneration and fibrosis at high levels of SphK1 (20-fold increase in SphK1 activity), whereas this is not observed at lower levels of SphK1 (5-fold increase in SphK1 activity) [126]. However, there is a contrary conclusion: SphK1 expression is significantly higher in cardiac fibroblasts than in cardiomyocytes. Under normal conditions, SphK1 is required for the proliferation of cardiac fibroblasts, but in the presence of myocardial hypoxia, SphK1 can exert anti-inflammatory effects and inhibit the development of cardiac fibrosis. Hence, SphK1 plays a dual regulatory role in cardiac physiology and pathology [110]. The above two studies suggest that SphK1 does play a regulatory role in cardiac fibrosis, but this role may be linked to the activity and expression of SphK1. Further research is necessary to investigate the mechanism by which SphK1 influences cardiac fibrosis.

In summary, S1P, S1PRs, and SphK are associated with the expression of several significant factors involved in the fibrotic pathway during the development of cardiac fibrosis. However, the molecular mechanisms governing the connection between the S1P signaling pathway and cardiac fibrosis remain unclear, as do the promoting or inhibiting effects on cardiac fibrosis under different conditions. Therefore, further ex vivo and in vivo studies are needed to determine whether these molecules in this pathway can serve as potential targets for the treatment of cardiac fibrosis in the future.

## Relationship between fatty acid carbon chain length in sphingolipids and cardiac fibrosis and atrial fibrillation

The length of fatty acid carbon chains in SLs has been found to have an impact on cardiac fibrosis and AF. To facilitate the description, this review categorizes SLs into four groups based on the length of the fatty acid carbon chains in the SLs as follows: (1) short-chain fatty acids: less than 6 carbons; (2) medium-chain fatty acids: 6–12 carbons; (3) long-chain fatty acids: 12–20 carbons; and (4) very long-chain fatty acids: greater than or equal to 20 carbons.

Very long-chain saturated fatty acids (VLSFAs) in circulation can originate from food or be synthesized endogenously. Small amounts of VLSFAs have been found in some nuts, seeds, and their extracted oils, with peanuts, macadamia nuts and rapeseed oil having the highest total VLSFAs content. Peanuts contain the highest content of 22:0 and 24:0 VLSFAs, while rapeseed oil contains the highest content of 20:0 VLSFAs. Sunflower seed oil has a higher proportion of 22:0 VLSFAs compared to other major commodity oils [136]. Other commodity oils, including corn oil, olive oil, soybean oil and safflower oil, contained small amounts of 20:0 VLSFAs. Short-term feeding trials have shown that supplementation with peanut [137] and macadamia nuts [138] can elevate circulating levels of VLSFAs.

**Fig. 7:**
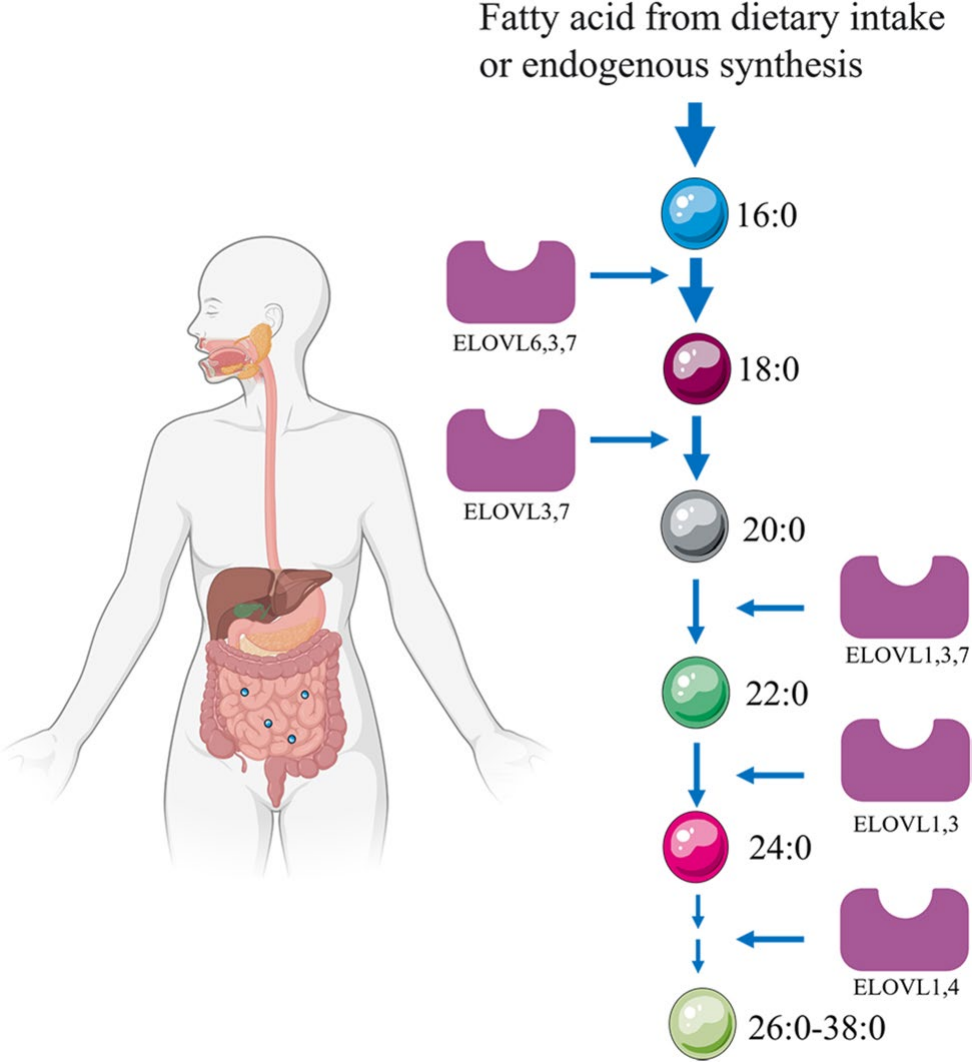
Endogenous synthesis of very long-chain saturated fatty acids (VLSFAs) [139]. VLSFAs are produced from 18:0 fatty acid chains catalyzed by the elongation of very long-chain fatty acids (ELOVL) family of enzymes. Seven ELOVL enzymes are present in vivo and overlap in the elongation steps they can catalyze. The 18:0 fatty acid chain can be derived from 16:0 or come from the diet. Unlike 18:0 and 16:0, VLSFAs are present in lower amounts, and only a small proportion of 18:0 is elongated to 20:0 and longer saturated fatty acids

The elongation of very long-chain fatty acids (ELOVL) enzyme family can catalyze the endogenous synthesis of VLSFAs from 18:0 fatty acid chains in the ER (Fig. 7) [139]. As the chain length of saturated fatty acids increases, the proportion of fatty acids absorbed in vivo decreases relatively, so the relative contribution to circulating VLSFAs levels by diet and metabolism may vary, but it is also unclear exactly what proportion is accounted for.

SLs containing saturated fatty acids with varying chain lengths exhibit distinct biological activities. Ceramide containing palmitic acid (Cer-16) promotes apoptosis, whereas ceramide containing VLSFAs prevents apoptosis and cardiomyocyte loss [140–143]. Apoptosis of cardiomyocytes in the atria heightens the risk of AF, with increased expression of apoptosis inducers in AF atrial tissue. In contrast, inhibiting the key apoptotic enzyme, caspase3, prevents electrical conduction disturbances and AF in the atria [87–89].

Jensen et al. found that ceramide and SLs containing palmitic acid (16:0) were linked to an increased risk of AF in a cardiovascular health study about the risk of AF, whereas ceramide and SLs containing very long chain saturated fatty acids were associated with a decreased risk of AF [144]. Fretts et al. reported that higher levels of several VLSFAs (arachidic [20:0], behenic [22:0], and lignoceric [24:0]) (arachidic [20:0], behenic [22:0], and lignoceric [24:0]) detected in phospholipids, including phosphoglycerides and sphingolipid fatty acids, were associated with a lower risk of AF [145]. Additionally, Lemaitre et al. systematically summarized that elevated levels of plasma VLSFAs were link to a lower risk of heart failure, AF, and mortality [139].Signori et al. found that increased concentrations of the very long-chain ceramide Cer (d18:1/24:0) reduced the risk of AF, while higher coffee intake was related to both increased blood Cer (d18:1/24:0) and a decreased risk of AF [146].

## Conclusion

SLs are crucial components of the plasma membrane in all vertebrate cells and play a role in regulating cellular functions such as cell proliferation, cell regulation, cell adhesion, cell migration, inflammatory responses, angiogenesis, and cell-cell interactions, and are thus associated with tissue fibrosis. Fibrosis of the heart, particularly atrial fibrosis, is the basis and most prominent feature of AF. Although there is still debate regarding whether atrial fibrosis is the cause or consequence of AF, recent research tends to suggest that some factors lead to atrial fibrosis and subsequently promote susceptibility to AF. When cardiac electrical conduction disorders occur, AF can be induced.

AF is one of the most common cardiac arrhythmias that has emerged as a significant public health concern. Globally, its prevalence continues to rise [7, 147]. The primary treatment for AF currently involves administering drugs to manage heart rate and rhythm, as well as using electrical (direct current and ablation therapy) cardioversion techniques to control ventricular rate or convert to sinus rhythm. Surgical interventions for symptom control are also utilized, and anticoagulants are frequently prescribed to prevent thromboembolic events arising from AF [9]. Unfortunately, no effective treatment for atrial fibrosis and permanent AF exists, and repairing fibrotic tissue to normal is challenging once fibrosis has set in. Therefore, the more effective strategy for treating this type of disease is currently to slow or prevent the development of fibrotic disease by obstructing upstream biological processes before fibrosis occurs in the tissues.

Numerous in vitro and in vivo studies have demonstrated that sphingolipid signaling pathways are involved in the occurrence and development of cardiac fibrosis. SLs, such as ceramide, C1P, and S1P can be involved in the regulation of cardiac fibrosis in a variety of ways. The length of the fatty acid chains in SLs can also influence their properties and participate in tissue fibrosis. Nonetheless, the mechanisms of sphingolipid signaling pathways in fibrosis and the interactions between related signaling pathways remain not entirely elucidated. The role of sphingosine in cardiac fibrosis has been poorly documented, and S1P and SphK1 might play varying roles in fibrosis under different conditions. Further research is necessary to clarify the mechanisms of sphingolipid signaling for fibrosis-related diseases, and interventions in this pathway may offer potential therapeutic options for fibrosis-related diseases. Furthermore, targeting the sphingolipid signaling pathway could be a promising approach for preventing or mitigating fibrotic diseases in the future.

## Data Availability

Not applicable.

## Nomenclature

SLs: Sphingolipids
AF: Atrial fibrillation
ECM: Extracellular matrix
S1P: Sphingosine-1-phosphate
C1P: Ceramide-1-phosphate
SM: Sphingomyelin
LCBs: Long-chain bases
PSLs: Phosphosphingolipids
GSLs: Glycosphingolipids
ER: Endoplasmic reticulum
CoA: Coenzyme-A
SPT: Serine palmitoyltransferase
ORMDLs: Orosomucoid-like proteins
KDSR: 3-ketodihydrosphingosine reductase
CerS: Ceramide synthase
SMases: Sphingomyelinases
CDases: Ceramidases
CERT: Ceramide transporter protein
SMS: Sphingomyelin synthase
DAG: Diacylglycerol
CPE: Ceramide phosphoethanolamine
ERK1/2: Extracellular signal-regulated kinase 1/2
CERK: Ceramide kinase
CGT: Ceramide galactosyltransferase
UDP-Gal: Uridine diphosphate galactose
GalCer: Galactosylceramide
GlcCer: Glucosylceramide
UGCG: UDP-glucose ceramide glucosyltransferase
CTGF: Connective tissue growth factor
Ang II: Angiotensin II
PDGF: Platelet-derived growth factor
TGF-β: Transforming growth factor-β
HF: Heart failure
BMDM: Bone marrow-derived macrophages
mTOR: Mammalian target of rapamycin
AA: Arachidonic acid
PLA2: Phospholipase A2
RLX: Relaxin
JNK: C-Jun n -terminal kinases
S1PRs: S1P receptors
SphK1: Sphingosine kinase 1
IL: Interleukin
VLSFAs: Very long-chain saturated fatty acids
ELOVL: Elongation of very long-chain fatty acids
